# Context effects on phoneme categorization in children with dyslexia

**DOI:** 10.1121/10.0002181

**Published:** 2020-10-19

**Authors:** Gabrielle E. O'Brien, Liesbeth Gijbels, Jason D. Yeatman

**Affiliations:** 1 Institute for Learning and Brain Sciences, University of Washington, Seattle, Washington 98105, USA; 2 Graduate School of Education, Stanford University, Stanford, California 94305, USA

## Abstract

Research shows that, on average, children with dyslexia behave less categorically in phoneme categorization tasks. This study investigates three subtle ways that struggling readers may perform differently than their typically developing peers in this experimental context: sensitivity to the frequency distribution from which speech tokens are drawn, bias induced by previous stimulus presentations, and fatigue during the course of the task. We replicate findings that reading skill is related to categorical labeling, but we do not find evidence that sensitivity to the stimulus frequency distribution, the influence of previous stimulus presentations, and a measure of task engagement differs in children with dyslexia. It is, therefore, unlikely that the reliable relationship between reading skill and categorical labeling is attributable to artifacts of the task design, abnormal neural encoding, or executive function. Rather, categorical labeling may index a general feature of linguistic development whose causal relationship to literacy remains to be ascertained.

## I. INTRODUCTION

It is well established that reading skill is correlated with performance on phoneme categorization tasks in which listeners are asked to categorize spoken syllables based on a single contrastive feature (Goswami *et al.*, 2002; Noordenbos and Serniclaes, 2015; O'Brien *et al.*, 2018; O'Brien *et al.*, 2019; Vandermosten *et al.*, 2010). However, the mechanism underlying the link between impaired processing of phonemes and developmental dyslexia remains unclear. While phonological awareness, the ability to identify and manipulate phonemes in speech, is one of the strongest predictors of dyslexia, there are several reasons to question that phonological processing is the “core deficit” that explains why all children with dyslexia struggle with learning to read. Some researchers have criticized this “core phonological deficit theory” on the grounds that not enough children could be accurately diagnosed on the basis of phonological awareness alone (Pennington *et al.*, 2012; Wolf and Bowers, 2000). Perhaps the most popular line of reasoning, though, is that children with dyslexia perform (on average) poorly on many measures of auditory processing, visual processing, working memory, and automaticity, which cannot be explained by a phonological deficit alone. These observations have motivated a new wave of research, searching for a more fundamental mechanism that might explain the myriad of deficits (including phonological awareness) that are associated with reading (dis)ability (Ahissar *et al.*, 2006; Jaffe-Dax *et al.*, 2017; Lieder *et al.*, 2019; Ziegler, 2008).

While some researchers have taken the perspective that individuals with dyslexia have fundamentally impaired auditory or visual processing (Goswami, 2011; Stein, 2018; Tallal *et al.*, 1996), the psychophysical literature on the whole is currently inconsistent with a homogeneous and uniform pattern of sensory impairment (Amitay *et al.*, 2002; Hämäläinen *et al.*, 2013; Rosen, 2003; Stuart *et al.*, 2006). Noting this, some researchers have argued that individuals with dyslexia are constrained not by sensory processing at a basic level but by the demands posed by psychophysical tasks (Ahissar, 2007; Ramus and Ahissar, 2012).

While the appeal to a domain-general mechanism could potentially explain the heterogeneity observed in the sensory processing literature, a consensus is yet to be reached regarding which particular aspects of the psychophysical tasks are the “bottleneck” in the performance. One candidate is attention and task vigilance; dyslexia is often comorbid with attention-deficit/hyperactivity disorder (ADHD; Germanò *et al.*, 2010; Light *et al.*, 1995; Stevenson *et al.*, 2005). In accord with this hypothesis, one previous study has shown that performance on “catch trials” tends to degrade faster over the course of a task in poor readers than do controls (Messaoud-Galusi *et al.*, 2011; see also Roach *et al.*, 2004, but note that Vandermosten *et al.*, 2018, reported null results in a similar design). Another candidate is statistical learning. Statistical learning was originally defined as “a way of extracting statistical regularities from the environment” (Saffran *et al.*, 1996). It has been proposed that individuals with dyslexia are less able than their typically developing peers to take advantage of regularities in their environment (Ahissar *et al.*, 2006; Banai and Ahissar, 2006; Gabay *et al.*, 2015; Lieder *et al.*, 2019). The statistical learning hypothesis is especially appealing because the process of learning to read involves forming connections between phonological and orthographic representations, which requires a learner to extract regularities from visual and auditory sequences (Ziegler and Goswami, 2005) and also to learn and automate the probabilistic relationship between a given letter and the phonemes it represents (Apfelbaum *et al.*, 2013). Distributional learning—sensitivity to the distribution from which stimuli are drawn—is known to be a key part of language acquisition in development (Maye *et al.*, 2002). Thus, there is growing interest in the possibility that individual differences in mechanisms such as sensitivity to environmental statistics, could explain the degraded performance across an array of experiments as well as a difficulty with learning to read.

The phoneme categorization task provides a reasonably reliable setting to explore how task performance may be differentially affected by task demands in struggling readers. Because it has been so extensively used, experimenters can have reasonable confidence that the key effect—shallower psychometric functions in struggling readers—is broadly replicable for many stop consonant continua (Noordenbos and Serniclaes, 2015; O'Brien *et al.*, 2018; O'Brien *et al.*, 2019). However, the mechanisms underlying the psychometric function shape may be difficult (if not impossible) to disambiguate as there are at least two plausible explanations for individual differences. First, individual differences in noise at the level of phonetic cue encoding could influence shape: increased noise around the category boundary will lead to a flatter function. Second, individual differences in categorization strategy must be considered. The optimal strategy—consistently applying the same label to every token with a phonetic cue above some threshold—would lead to a steep psychometric function, whereas probability matching based on the statistics of the cue distribution (detailed in Clayards *et al.*, 2008) would lead to a shallower function. It is unclear which strategy children might use in this experimental context or which mechanism is most relevant to children's performance on the task. While these limitations do not invalidate the task as a probe of some dimension of speech perception related to literacy skill, they are worth bearing in mind.

Previously, we showed that reduced categorization in struggling readers is somewhat influenced by the working memory demands of the task but could not be entirely explained by task difficulty: irrespective of the task-difficulty, we found a correlation between reading skill and task performance (O'Brien *et al.*, 2018). We now investigate several other aspects of task performance to clarify the extent to which the phoneme categorization-reading relationship depends on specific experimental conditions.

We first consider the effects of varying the stimulus distribution from which speech tokens are drawn. Typically, in categorization experiments, stimuli are drawn from a uniform distribution. Adults without reading disability show sensitivity to the stimulus distribution in the categorization task (Clayards *et al.*, 2008): the task elicits more categorical behavior when stimuli are drawn from narrow bimodal distributions than when drawn from broad distributions. Recently, work by Vandermosten *et al.* (2018) suggested that children with dyslexia were, on average, less able to utilize distributional cues to learn a non-native speech contrast. In the present study, we examine how children aged 8–12 years performed on a categorical phoneme labeling task with two conditions: a bimodal and uniform distribution of native speech tokens (note that although Clayards *et al.*, 2008, compared two bimodal distributions of various widths, a uniform distribution is equal to an infinitely wide bimodal distribution). This result is of interest for two reasons. First, we hope to better characterize the matter of distributional sensitivity in struggling readers, which is largely unsettled in the literature. Second, studying nonuniform stimulus distributions may bring the speech categorization task closer to representing ethological conditions. In natural speech, utterances are typically “drawn” from a structured distribution—it is this structure that may enable children to learn categories in the first place (McMurray *et al.*, 2009). If struggling readers are indeed more categorical when presented with stimuli from bimodal versus uniform distributions, that suggests their ability to perceive speech in naturalistic conditions may be less impaired than many researchers argue on the basis of the categorical labeling task (Noordenbos and Serniclaes, 2015).

Next, we explore how the immediate context of recently presented speech tokens affects judgments about the identity of the current stimulus (e.g., after a clear phoneme exemplar is heard, a listener might be more likely to judge an ambiguous speech sound as representing a different category). Considering how recent stimulus presentations influence performance is of interest for several reasons: it addresses longstanding claims that individuals with dyslexia struggle when stimuli are presented sequentially (Tallal, 1980) or that they show abnormal stimulus adaptation and faster implicit memory decay (Ahissar *et al.*, 2006; Jaffe-Dax *et al.*, 2017; Perrachione *et al.*, 2016). Finally, we look for hallmarks of fatigue and disengagement in our participant's responses by examining changes in task performance over the duration of the experiment. In line with previous work, we find that there is a moderate relationship between phoneme categorization and reading skill; this relationship cannot be attributed to (1) the stimulus distribution, (2) stimulus recency effects, or (3) task disengagement. Thus, we conclude that some people with dyslexia have difficulties categorizing speech sounds and this deficit, though likely not universal, is not an artifact of experimental conditions, such as the distribution, order, and duration of the experiment.

## II. METHODS

### A. Participants

A total of 62 native English-speaking children aged 8–12 years were recruited for the study. Children without known auditory disorders were recruited from a database of volunteers in the Seattle area (University of Washington Reading and Dyslexia Research Database^1^). Parents and/or legal guardians of all participants provided written informed consent under the University of Washington Institutional Review Board protocol. All subjects demonstrated normal or corrected-to-normal vision. Participants were tested on a battery of cognitive and literacy assessments, including the Woodcock-Johnson IV (WJ-IV) Letter-Word Identification and Word Attack subtests, the Test of Word Reading Efficiency (TOWRE), and the Weschler Abbreviated Scale of Intelligence (WASI). All participants underwent a hearing screening to ensure pure tone detection at octave frequencies between 500 and 8000 Hz in both ears at 25 dB hearing level (HL) or better.

### B. Demographics

Here, we present analyses of task performance where reading skill is treated as either a continuous or discrete group variable. It has been reasonably established that reading skill is best modeled as a continuous variable with no clear demarcation between readers who are below-average and readers who are dyslexic (Shaywitz *et al.*, 1992). Many results on phoneme categorization published so far (Goswami *et al.*, 2002; O'Brien *et al.*, 2018; O'Brien *et al.*, 2019; Vandermosten *et al.*, 2011) support the perspective that there is a continuous relationship between task performance and reading skill. However, for completeness and ease of comparison with existing literature on dyslexia, we also provide group-level analyses.

Reading skill was summarized in a composite variable: as both the Woodcock-Johnson Basic Reading Skill measure (WJ-BRS; a composite of word attack and letter word identification subtests) and the TOWRE index (a composite of the sight word efficiency and phonemic decoding efficiency subtests) are scored on the same standardized scale [mean = 100, standard deviation (SD) = 15], a composite reading skill measure was created by averaging the two metrics for each participant. Using a composite of both measures as the criterion improves the reliability of our group assignments because they are highly correlated measures (r=0.877,p<0.001, in our sample). Participants were assigned to the dyslexia group if their composite reading score was at least 1 SD below the population mean (i.e., <85). Six participants had reading scores above the 1 SD cutoff but a parental report of dyslexia; as in our previous work (O'Brien *et al.*, 2019), these participants were excluded from the control group for group-level comparisons but are included in all other statistical analyses and data. The scores for these six participants fell between 86.5 and 92; they may represent children who, at one point, met criteria for reading disability but have since been remediated into the low-end of the typical range. Another possibility is that these children struggled on measures that we did not consider here, but would be of interest to the diagnosing professional. We cannot be certain as there is no standard for diagnosis among professionals in our area that we can relate to the reading measures assessed here.

Additionally, all subjects were required to have nonverbal intelligence quotient (IQ) and full-scale IQ (WASI matrix reasoning and FS-2 scores, respectively) no less than 1 SD below the population mean (as in O'Brien *et al.*, 2018; O'Brien *et al.*, 2019); three subjects were below this cutoff and excluded from further analysis. This left a total of 59 participants eligible for the study based on their cognitive characteristics, 53 of which could be confidently categorized as dyslexic or control for the purpose of group-level comparisons.

There were 24 subjects in the dyslexic group (13 male) and 29 subjects in the control group (14 male). The mean age and SD were 9.5 yr (1.4 yr) and 10.0 yr (1.5 yr), respectively, in the dyslexic and control groups; the difference in age was not significant (Kruskal-Wallis rank sum test, H(2)=3.573,p=0.168), although we noted that there was a small correlation between age and reading skill (r=0.253,p=0.053). Importantly, we tested age as a covariate in our exploratory secondary models. We did not exclude participants with ADHD diagnoses from the study because ADHD is highly comorbid with dyslexia (Germanò *et al.*, 2010). Indeed, research suggests that there is little validity in distinguishing children with dyslexia and a secondary comorbid diagnosis (Boada *et al.*, 2012; Peters and Ansari, 2019). Therefore, we accounted for ADHD diagnosis in our exploratory covariate analysis. Of 59 total participants, 13 had a formal diagnosis of ADHD: 7 in the dyslexic group and 4 in the control group. The difference in prevalence of ADHD across groups was not significant (H(1)=1.851,p=0.174).

Table I shows group comparisons on measures of reading and cognitive skills. Note that IQ (either measured as full-scale or nonverbal) differed by group. While we were not concerned that low IQ prevented any subject from understanding the task because low IQ was an exclusion criterion, we included nonverbal IQ as a covariate in our statistical analyses.

**TABLE I.** Summary statistics and group differences on demographic and behavioral measures.

	Control	Dyslexic	
	n= 29	n= 24	
	14 male, 15 female	13 male, 11 female	Significance
WASI-III			
FS-2	123.9 (11.3)	101.8 (13.1)	<0.001
Nonverbal IQ	60.5 (8.6)	50.3 (7.8)	<0.001
WJ-IV			
Basic reading score	115.3 (9.8)	79.8 (7.2)	<0.001
Nonword	114.4 (12.3)	87.8 (10)	<0.001
Real word	114.1 (8.7)	74.8 (10.3)	<0.001
TOWRE 2			
TOWRE index	105.2 (11.1)	69.5 (7)	<0.001
Nonword	104.6 (12)	72.9 (8.2)	<0.001
Real word	105.5 (11.3)	69.2 (9.3)	<0.001
CTOPP 2			
Phonological awareness	102.8 (13.3)	91.6 (11.4)	0.011
Phonological memory	100.5 (13.8)	88.2 (12.1)	0.002
Rapid naming	99.4 (11.4)	83.7 (9.8)	<0.001

### C. Stimuli

A seven-step /ba/∼/da/ speech continuum was created using Praat version 6.0.37 (Boersma and Weenink, 2020). Synthesis of the continuum followed the procedure described in O'Brien *et al.* (2018), using linear predictive coding to alter the formant contours of a naturally produced /ba/ token. In the /ba/∼/da/ continuum, the starting frequency of the second vowel formant (F2) transition was varied. In brief, the seven speech tokens were identical except for their F2 formant contour. All tokens were resynthesized from a /ba/ utterance spoken by a male American English speaker. The starting frequency of F2 was varied in seven linearly spaced steps from 1085 Hz (/ba/) to 1460 Hz (/da/). F2 followed a linear ramp to a terminal value of 1225 Hz over the course of 100 ms at which point the steady-state portion of the vowel was maintained for 250 ms.

### D. Procedure

Stimulus presentation and participant response collection was managed with PsychToolbox for matlab (Brainard, 1997). Auditory stimuli were presented at 75 dB sound pressure level (SPL) via circumaural headphones (Sennheiser HD 600, Wedemark, Germany). Children were trained to associate sounds from the two speech continua with animal cartoons on the left and right sides of the screen and indicate their answers by pressing the right or left arrow key. Large text labels were provided over each animal cartoon (“Ba” on the left side and “Da” on the right) so that participants did not have to memorize the animal associated with each sound. Throughout all blocks, each cartoon was always associated with the same stimulus end point.

Participants first completed a practice round consisting of ten presentations, five of each continuum end point, with feedback on each trial. Participants were allowed to repeat the practice round up to three times until they had achieved at least 75% accuracy. All participants were able to meet this minimum standard.

The main task was presented in two parts, one in which the stimuli were drawn from a uniform frequency distribution and another in which they were drawn from a bimodal distribution. In the unimodal condition, all stimuli were presented 15 times. In the bimodal condition, the presentation frequency was greatest at the continuum end points and least in the center of the continuum (see Table II).

**TABLE II.** Stimulus presentation frequency in bimodal condition.

Stimulus	1	2	3	4	5	6	7
Frequency	52	34	14	10	14	34	52

Because we were interested in exploring the effects of recently presented stimuli on judgments about the current stimulus, we used a “random but frozen” list of stimuli. This means that we randomly generated the order in which stimuli would be presented in each condition, but every participant was tested with this fixed stimulus order. This reduced one source of variability across subjects so that we could perform more targeted investigations about how recent stimulus presentations differentially affect strong and poor readers.

In each condition (uniform or bimodal frequency distribution), participants heard a total of 210 speech sounds. After every 35 stimulus presentations, a quick optional break was presented. Between the two test conditions, reading assessments were performed. If a participant did not already have an IQ measure on file from a previous laboratory visit, the WASI-III was also administered.

Note that three subjects did not wish to complete the task and opted to quit part of the way through; one such participant came from the control group and the other two participants were from the dyslexic group. Their data were omitted from the study. Complete data were, therefore, collected from a total of 56 participants (28 control, 22 dyslexic, and 6 not categorized).

Seven participants completed the uniform condition first and 49 completed the bimodal condition first. The reason for this discrepancy is that during data collection for the first 15 subjects, we alternated which distribution was presented first. After data were collected for these subjects, we were surprised to see little evidence that participants behaved differently in either condition—particularly because of the positive evidence from two published studies (Clayards *et al.*, 2008; Vandermosten *et al.*, 2018) and our own pilot data in six subjects, which appeared consistent with an effect of stimulus distribution. We, thus, changed to a policy of always providing the bimodal distribution first, wary that initial exposure to the uniform distribution could affect category learning in subsequent conditions. We were unable to detect any significant differences between task performance in these individuals and the remainder of the cohort. Psychometric function slope did not significantly differ by group [β=−0.142, standard error (SE) = 0.365, *p* = 0.698], nor was there a significant interaction between the order distributions were presented and slope in each condition (β=0.294, SE = 0.485, *p* = 0.547). Based on this evidence, we retained these seven subjects in the data set.

### E. Psychometric curve fitting

We used the matlab toolbox Psignifit 4.0 (The MathWorks, Natick, MA) to fit psychometric functions. The fitting routine optimized the fit of a logistic curve function with four parameters modeling the upper and lower asymptotes, width of the logistic function, and the category boundary. The width of the logistic function was transformed to the slope at the category boundary value (the estimated point on the continuum where 50% of tokens are labeled Da) to give a standardized measure of psychometric function slope.

Psignifit uses a Bayesian framework to optimize parameter estimates not only according to likelihood of generating a given set of behavioral responses but also with regard to prior distributions of each parameter. In the case of psychometric function fitting, where the number of presentations of each stimulus is often relatively low, inappropriate priors can have an outsized influence on parameter estimates—particularly, as we and many others have summarized elsewhere, when it comes to estimates of the slope and asymptotes. We, therefore, used priors identical to a previous study using this /ba/∼/da/ continuum (O'Brien *et al.*, 2019): the asymptotic priors were modeled as a uniform distribution on the range [0,0.10]. In other words, the lower and upper asymptotic parameters could vary freely in the range [0,0.10] to give a lower asymptote between 0% and 10% and an upper asymptote between 90% and100%. This prior width was chosen on the basis of tenfold cross-validation over the data set to determine the psychometric fitting parameters that best predicted the participant's decisions on held-out trials (see O'Brien *et al.*, 2018; O'Brien *et al.*, 2019, for further details).

To ensure the validity of psychometric function parameter estimates, we excluded any psychometric functions that could not be fit with a category boundary between continuum steps 1–7. Only one psychometric function (produced by a subject in the dyslexic group presented with a uniform stimulus distribution) was excluded on these grounds.

We checked for correlations between reading skill and several metrics of psychometric function fit. The correlation between reading skill and sum of squared residuals (averaged over each participant's two psychometric function fits) was not significant (*r* = −0.185, *p* = 0.092). Likewise, deviance of the fits was not significantly associated with reading skill (*r* = −0.076, *p* = 0.41).

### F. Statistical analysis of parameter estimates

After we fit psychometric functions for each subject in each condition, we used a series of generalized linear mixed models to determine the relationship between reading ability, the frequency distribution from which stimuli are drawn, and four dependent measures. These dependent measures were estimates of task performance based on behavioral responses: (1) psychometric function slope, (2) asymptote, (3) category boundary, and (3) a composite measure of psychometric function shape.

Each participant's average asymptote was determined by averaging the upper and lower asymptote estimates of a given function (i.e., their deviations from zero and one, respectively). The composite measure PC1 was constructed from a principal components analysis on the four parameters of each psychometric function collected in the study. The first principal component captured 46.1% of variance in the four parameters and was defined by the following linear weights: category boundary, −0.385; slope, 0.479; upper asymptote, −0.504; lower asymptote, −0.607.

Linear modeling was performed with the lme4 library for *R*. For each dependent measure, fixed-effect predictors with sum coding were used for the distribution (uniform or bimodal) variable. Reading ability was entered as a continuous fixed-effect predictor except where otherwise stated.

**FIG. 1.**
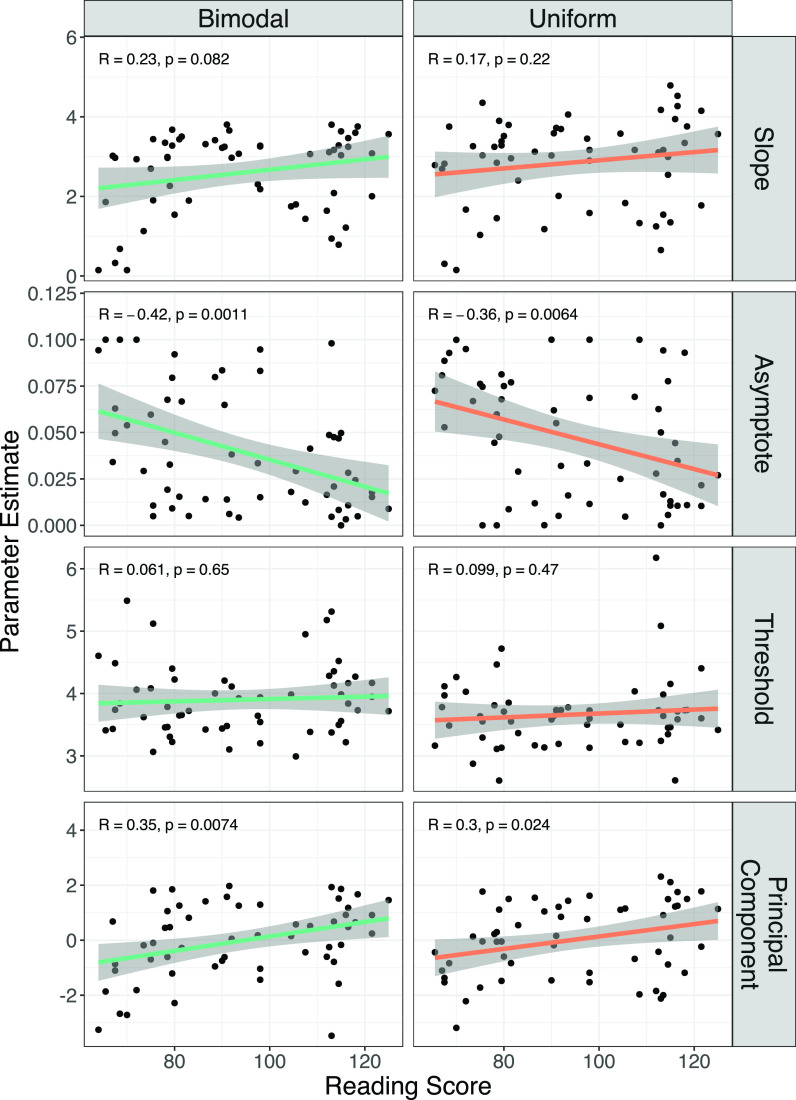
(Color online) Plots of model psychometric function parameter estimates versus reading score. Each point corresponds to parameter estimates for one subject in one condition (bimodal or uniform distribution). Lines indicate the best fit regression line with 95% confidence intervals in shaded regions.

We tested a core model,

parameter∼reading∗distribution+(1|subject ID), (1)

where *parameter* was the psychometric parameter of interest: slope, lapse, category boundary, or PC1. We also tested the additions of three additional “nuisance” predictors to this core model: the presence/absence of ADHD diagnosis (treatment coding), age (continuous predictor), and non-verbal IQ (WASI-III matrix reasoning score; continuous predictor). The core modeling results are reported in the text, and the effects of the individual nuisance predictors are reported in Fig. 1 and Table III.

**TABLE III.** Core models of psychometric function parameters.

	*β*	SE	*p*
Slope			
Reading skill	0.224	0.118	0.062
Distribution	0.238	0.161	0.15
Reading skill ∗ distribution	−0.026	0.163	0.87
Asymptote			
Reading skill	−0.013	0.004	<0.001
Distribution	0.008	0.004	0.049
Reading skill ∗ distribution	0.001	0.004	0.89
Category boundary			
Reading skill	0.038	0.071	0.59
Distribution	−0.230	0.064	<0.001
Reading skill ∗ distribution	0.007	0.064	0.92
PC1			
Reading skill	0.475	0.163	0.005
Distribution	−0.015	0.128	0.91
Reading skill ∗ distribution	−0.013	0.129	0.92

### G. Data availability statement

Data are available immediately in a GitHub repository hosted by the laboratory.^2^

## III. RESULTS

As expected on the basis of previous studies, we found relationships between reading skill and psychometric function shape (Noordenbos and Serniclaes, 2015; O'Brien *et al.*, 2018; O'Brien *et al.*, 2019; Vandermosten *et al.*, 2010). In Fig. 1, we can see that some psychometric parameters were correlated with reading ability, most notably the asymptote and PC1. Category boundary (the estimated point on the continuum with 50% of tokens labeled Da) was not significantly correlated with reading ability in either the uniform or bimodal condition.

We confirmed this with a generalized linear mixed model analysis, first, with regard to the relationship between reading ability and psychometric slope. In our core model of slope (Table III), reading skill was associated with a sharper slope as expected, although this effect did not reach the threshold of significance (β=0.224, SE = 0.118, *p* = 0.062). Note that the main effect of distribution (β=0.238, SE = 0.161, *p* = 0.15) and the interaction of distribution and reading ability (β=−0.026, SE = 0.163, *p* = 0.87) were not significant. No nuisance variable proved to have a significant effect on slope (see Table IV).

**TABLE IV.** Effect of nuisance variables on slope.

	*β*	SE	*p*
Age			
Reading skill	0.174	0.120	0.15
Distribution	0.237	0.161	0.15
Age	0.134	0.082	0.11
Reading skill ∗ distribution	−0.024	0.162	0.88
ADHD			
Reading skill	0.212	0.120	0.083
Distribution	0.238	0.161	0.15
ADHD	−0.191	0.301	0.53
Reading skill ∗ distribution	−0.027	0.163	0.87
Nonverbal IQ			
Reading skill	0.200	0.139	0.16
Distribution	0.238	0.161	0.15
Nonverbal IQ	0.047	0.140	0.74
Reading skill ∗ distribution	−0.026	0.163	0.88

Thus, we did not detect a significant relationship between psychometric slope and the frequency distribution from which stimuli were drawn. Moreover, we did not find evidence supporting the hypothesized interaction between reading skill and experimental condition (bimodal or uniform distribution).

Similarly, we tested the core model as a predictor of the asymptote and found a significant main effect of reading skill (β=−0.013, SE = 0.004, *p* < 0.001). There was also a modest main effect of distribution on the asymptote; the uniform distribution was associated with an 0.008 point greater asymptote than the bimodal distribution (SE = 0.004, *p* = 0.049). While this effect is small, it is in the direction we would expect if the bimodal distribution had a stabilizing effect on phoneme categories in most participants (at least, more reliable labeling of the clear category exemplars at the end points of the continuum). Importantly, the interaction of the asymptote and reading skill was not significant. Of the nuisance variables, only age was significant (see Table V). Even when age was included in the model, the main effect of reading skill remained significant.

**TABLE V.** Effect of nuisance variables on asymptote.

	*β*	SE	*p*
Age			
Reading skill	−0.011	0.003	0.003
Distribution	0.008	0.004	0.048
Age	−0.006	0.002	0.010
Reading skill ∗ distribution	0.001	0.004	0.90
ADHD			
Reading skill	−0.012	0.004	<0.001
Distribution	0.008	0.004	0.050
ADHD	0.012	0.009	0.17
Reading skill ∗ distribution	0.001	0.004	0.88
Nonverbal IQ			
Reading skill	−0.013	0.004	0.003
Distribution	0.008	0.004	0.049
Nonverbal IQ	0.000	0.004	0.94
Reading skill ∗ distribution	0.001	0.004	0.89

Next, we modeled category boundary. Reading skill and the interaction of reading skill and distribution were both insignificant predictors. There was a significant main effect of distribution on category boundary; in the bimodal condition, participants, on average, tended to have a higher category boundary than in the uniform condition. In other words, they were slightly biased to label sounds as ba in the bimodal condition relative to the uniform condition. The effect is modest (an average shift of approximately 1/4 of a step on the continuum). We had not hypothesized that the category boundary would shift with the stimulus distribution, and it is important to note that having a higher or lower category boundary is not generally considered *better* for speech perception. As such, this finding should be considered *post hoc*. Still, the associated *p*-value would pass most standard corrections for multiple corrections (β=−0.230, SE = 0.064, p≤0.001). No nuisance variable was a significant predictor of the category boundary (see Table VI).

**TABLE VI.** Effect of nuisance variables on category boundary.

	*β*	SE	*p*
Age			
Reading skill	0.054	0.073	0.47
Distribution	−0.229	0.064	<0.001
Age	−0.042	0.050	0.41
Reading skill ∗ distribution	0.006	0.064	0.92
ADHD			
Reading skill	0.053	0.071	0.46
Distribution	−0.230	0.064	<0.001
ADHD	0.236	0.179	0.19
Reading skill ∗ distribution	0.007	0.064	0.91
Nonverbal IQ			
Reading skill	0.069	0.083	0.41
Distribution	−0.230	0.064	<0.001
Nonverbal IQ	−0.061	0.084	0.47
Reading skill ∗ distribution	0.007	0.064	0.92

Last, considering PC1 as the dependent variable, reading skill was a significant predictor of PC1 (Table III). Neither distribution nor the interaction of reading skill and distribution were significant predictors. Of the nuisance variables, only age was associated with a significant effect; after accounting for age, the effect of reading skill was still significant (see Table VII).

**TABLE VII.** Effect of nuisance variables on PC1.

	*β*	SE	*p*
Age			
Reading skill	0.367	0.161	0.026
Distribution	−0.016	0.127	0.90
Age	0.285	0.110	0.013
Reading skill ∗ Distribution	−0.013	0.129	0.92
ADHD			
Reading skill	0.437	0.163	0.010
Distribution	−0.014	0.128	0.91
ADHD	−0.605	0.411	0.15
Reading skill ∗ distribution	−0.015	0.129	0.91
Nonverbal IQ			
Reading skill	0.438	0.192	0.027
Distribution	−0.015	0.128	0.90
Nonverbal IQ	0.072	0.194	0.71
Reading skill ∗ distribution	−0.013	0.129	0.92

Taken as a whole, we detected only minor effects of the stimulus distribution on the asymptote and category boundary. Neither of these effects was hypothesized from the outset. Most importantly, we did not detect that any effect of the stimulus distribution on any psychometric parameter meaningfully varied with reading skill.

We also computed the Bayes factor (BF), which describes the ratio of the likelihoods that our data set was generated by either of the two models: *H*_0_, the null model with no interaction between reading skill and stimulus distribution, and *H*_1_, the model containing the hypothesized interaction (Kass and Raftery, 1995). While a *p*-value describes the likelihood of rejecting the null, the BF estimates which model is more likely given the data (Wagenmakers, 2007).

We used an estimation of the BF from the Bayesian information criterion (BIC; Wagenmakers, 2007),

$${\text{BF}}_{01}\approx \;\text{exp}\;\left(\frac{\text{BIC}(H_{1})-\text{BIC}(H_{0})}{2}\right).$$ (2)

In this case, the model *H*_0_ is defined as

parameter∼reading+distribution+(1|subject  ID), (3)

where the dependent variable *parameter* is a parameter of the fitted psychometric functions—slope, asymptote, category boundary, or PC1. Similarly, the model *H*_1_ is defined as

parameter∼reading+distribution+reading∗distribution+(1|subject  ID). (4)

For the slope, ${\text{BF}}_{01}=25.6$; for the asymptote, ${\text{BF}}_{01}=1007.6$; for the category boundary, ${\text{BF}}_{01}=65.4$ and for PC1, ${\text{BF}}_{01}=32.6$. In all cases, the BF indicates considerably stronger evidence for the null model (i.e., no interaction of reading skill and stimulus distribution). By standard BF reporting, these results would be considered strong to very strong evidence for the null.

For completeness, we also tested the interaction of reading skill and stimulus distribution with reading skill treated as a categorical variable (dyslexic versus control). A mixed effects analysis of variance (ANOVA) with a random effect of subject was used to evaluate the interaction term, using the Kenwards-Rogers estimation of degrees of freedom. The interaction was not significant in a model of the slope [F(1,48.3)=0.051,p=0.821], the asymptote [F(1,62.2)=0.005,p=0.942], the category boundary [F(1,47.9)=0.46,p=0.830], or PC1 [F(1,48.0)=0.0001,p=0.991]. The estimated Cohen's *d* for the separation of slope by group was 0.43 with a 95% confidence interval ranging from −0.15 to 1.00; this range is consistent with the average effect size in a meta-analysis of categorical labeling studies (Noordenbos and Serniclaes, 2015). For separation of the asymptote by group, *d* = 0.87 (CI =[0.27,1.47]), by category boundary, *d* = 0.08 (CI = [−0.50,0.65], and for separation of PC1 by group, *d* = 0.67 (CI =[0.08,1.27]).

Altogether, our analyses indicate that from the behavioral responses alone, there is little evidence that poor readers and strong readers are differentially affected by the stimulus distribution of the categorical labeling task. Our findings are somewhat complicated by the fact that we did not find clear evidence for a robust effect of stimulus distribution on categorical behavior; the small main effects on category boundary and asymptote are difficult to interpret and not expected from prior literature. To the extent that our data can speak to the effects of stimulus distribution on task performance, though, our results do not support a relationship between dyslexia and altered distributional sensitivity on this particular task.

### A. Effects of recent stimulus presentations on phoneme labeling

Because we collected 420 responses per individual, our data set may provide sufficient power to examine stimulus recency effects. To explore this possibility, we employed the modeling approach of Lieder *et al.* (2019), which uses generalized linear models (GLMs) to investigate how recent stimulus presentations affect the judgment of the current stimulus' identity.

For every stimulus presentation in the data set, we determined the identity of the preceding four stimuli. As in Lieder *et al.* (2019), we adopt the following notation.

Let *d*_0_ be the stimulus steps (1–7) of the current stimulus presentation, *t*. Then, *d*_1_ is the difference in steps between *d*_0_ and the stimulus presented at trial *t* – 1. Similarly, *d*_2_ is the difference in steps between *d*_0_ and the stimulus presented at trial *t* – 2, and so on for values *d*_3_ and *d*_4_.

The mixed effects GLM specifying the relationship between the label assigned to the current stimulus presentation and the recent presentations is as follows:

$$\text{response}=f(\beta_{0}+\beta_{1}d_{0}+\beta_{2}d_{1}+\beta_{3}d_{2}+\beta_{4}d_{3}+\beta_{5}d_{4}+(1|\text{subject}\;\text{ID})),$$ (5)

where *f* is the probit link function, *β* coefficients are linear weights to be estimated, *d*_0_ represents the continuum step of the current stimulus presentation, and (1|subject  ID) is a random intercept for subject. The probit was chosen as the link function because the dependent variable, *response*, is binomially distributed—i.e., participants decided whether a sound was da or not. We note that the probit function contains only two parameters that variously adjust the slope and category boundary of a sigmoid and, therefore, differences in the asymptote would have an effect on the estimated slope.

**FIG. 2.**
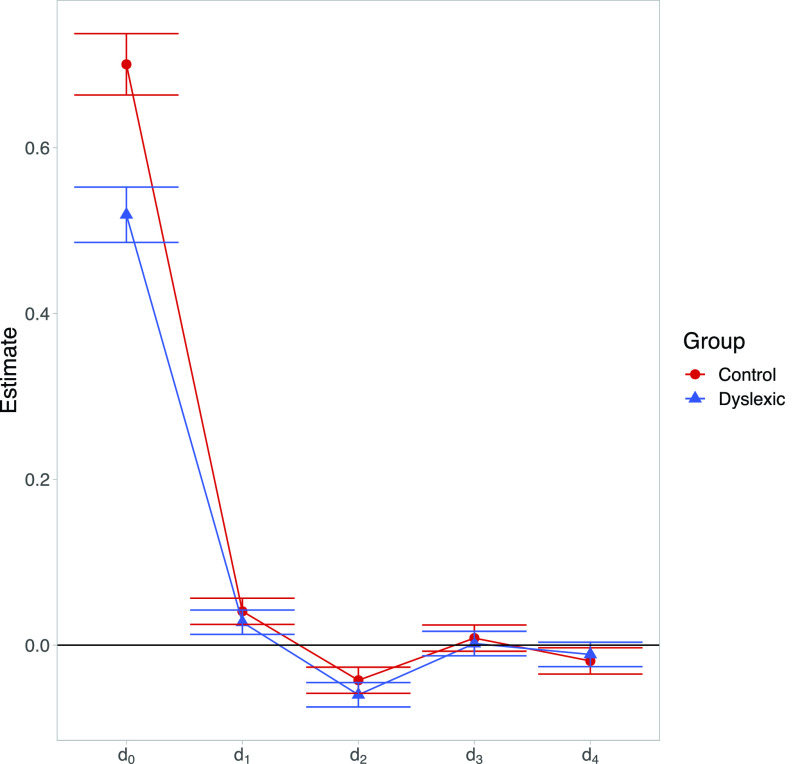
(Color online) Estimates of coefficients from a mixed effects generalized linear model (GLM) fit to group behavioral data. Bars indicate the 95% confidence interval surrounding a given estimate.

For the purpose of illustrating this approach clearly, we begin with an exploration of group differences and then move on to a model where reading skill is treated as a continuous variable. We first fit a mixed effects GLM to the responses of each group (control and dyslexic). The estimated coefficients are compared in Fig. 2.

We can immediately see that stimulus recency effects exist and (*d*_1_–*d*_4_) are quite similar between groups. The coefficient that differs by group is the weighting of *d*_0_—in other words, the mixed effects GLM estimates that the probit slope is lower in the dyslexic group even when recent stimulus presentations are accounted for.

Having visualized the group-level differences, we follow up with a treatment of reading skill as a continuous measure in the GLM. On the basis of our initial exploration, we drop the terms *d*_3_ and *d*_4_ from the model as the standard errors for these point estimates suggest we are underpowered to detect recency effects at this time scale.

First, we consider the mixed effects GLM containing main effects of *d*_1_, *d*_2_, *d*_0_, and reading skill, plus an interaction of *d*_0_ and reading skill. We hypothesized a significant interaction between reading skill and *d*_0_ on the basis of the previous group-level model. Indeed, the interaction, as well as the stimulus recency terms, were all highly significant (Table VIII).

**TABLE VIII.** Hypothesized model of behavioral response.

	*β*	SE	*p*
Reading skill	0.002	0.034	0.95
*d*_0_ ∗ Reading skill	0.132	0.006	<0.001
*d*_0_	0.633	0.010	<0.001
*d*_1_	0.035	0.005	<0.001
*d*_2_	−0.049	0.005	<0.001

We also tested augmenting our hypothesized model to include an interaction between reading skill and stimulus recency terms *d*_1_ and *d*_2_. Adding a *d*_1_ ∗ reading skill interaction increased the Akaike Information Criterion (AIC) from 15 868.8 to 15 869.0 and increased the BIC from 15 925.0 to 15 933.3, and the new interaction term was not significant (β=0.007, SE = 0.005, *p* = 0.182). We, again, computed the BF to assess the relative evidence for the presence of an interaction; we estimated ${\text{BF}}_{01}=61.9$, consistent with strong evidence for the null. Considering a *d*_2_ ∗ reading skill interaction term fared no better: the interaction was not significant (β=0.0007, SE = 0.005, *p* = 0.896), and AIC and BIC increased (to 15 870.8 and 15 935.0, respectively) compared to the simpler model. Again, the BF indicated strong evidence for the null with ${\text{BF}}_{01}=149.6$.

From this investigation of stimulus recency effects, we find that we are able to detect highly significant effects of the last two stimulus presentations on judgments of the current stimulus' identity. We did not detect a significant interaction of reading skill and the influence of previous stimulus presentations, and Bayesian analysis provides evidence against the presence of an interaction. In all, the model upholds the interpretation that psychometric functions are steeper in stronger readers regardless of the context in which each stimulus presentation occurs.

Last, we performed an analysis to characterize the mechanism by which recently presented stimuli influence judgments of the current stimulus. We hypothesized that if the current stimulus was ambiguous—i.e., it was drawn from the center of the /ba/∼/da/ continuum—then the influence of the previous stimulus would be greatest. In other words, listeners might make greater use of the contrast between the current and previous stimuli when the current stimulus is ambiguous than when the current stimulus is a clear category exemplar. We tested this hypothesis with another mixed effects GLM. First, we created a new binary feature that distinguishes stimuli drawn from the center of the continuum versus stimuli drawn from the end points,

$$\text{ambiguous}=\{\begin{array}{cc} 0, & \text{if}\;d_{0}\in [\text{steps}\;\;1,2,6,7],\\ 1, & \text{otherwise}. \end{array}$$

We then tested the model

$$\text{response}=f(\beta_{0}+\beta_{1}d_{0}+\beta_{2}d_{1}*\text{ambiguous}+(1|\text{subject}\;\text{ID})),$$ (6)

where *f* is a probit function as in Eq. (5). We were specifically interested in the interaction of *d*_1_ and *ambiguous*: a significant interaction indicates that the magnitude of the difference between the current and past stimulus depends on whether the current stimulus is a category exemplar or not. As expected, the interaction of *d*_1_ and *ambiguous* was significant (β=0.085, SE =0.007,p<0.001). This analysis upholds the intuition that previous trials influence the present judgment by providing a contrast by which to judge ambiguous stimuli. If this is indeed the primary mechanism by which stimulus recency effects influence performance on the categorization task, then our study is not alone in finding a lack of interaction between reading skill and such contextual effects: Blomert and Mitterer (2004) also found no evidence for context effects at several linguistic scales in a phoneme labeling task like ours.

### B. Quantifying fatigue during the task

The relatively large number of trials collected per subject allows us to revisit an analysis proposed by Messaoud-Galusi *et al.* (2011) to determine whether poor readers show precipitous declines in task performance as the study goes on. If poor readers become fatigued or distracted at a faster rate than strong readers do, that could explain overall differences in task performance.

**FIG. 3.**
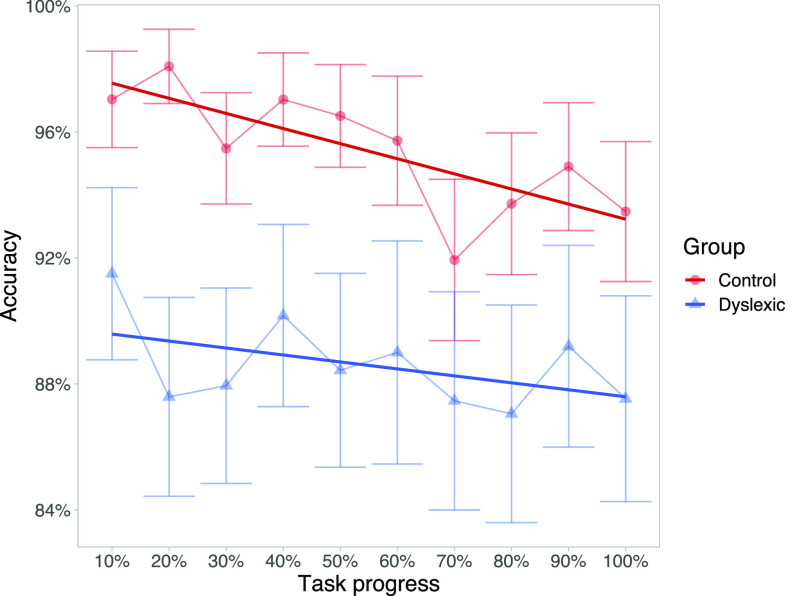
(Color online) Each point represents the average accuracy within a group in a certain interval of the task. Error bars mark the 95% confidence interval of the mean. The *x* axis marks progress through the task: 10% marks the first 21 of 210 trials, 20% marks trials 22–42, and so on. Dark lines indicate the best fit regression line.

To this end, we modeled the probability of correctly labeling a clear ba or da exemplar as a function of the trial number and reading skill (Fig. 3). Note that clear category exemplars are stimuli drawn from the two ends of the continuum (steps 1 and 7). Having already established that poor readers produce shallower psychometric functions overall, we should expect that the probability of correctly labeling these tokens will be lower overall in poor readers. If an interaction of trial number and reading skill is found to be significant, that would suggest task fatigue occurs differentially across the spectrum of reading skill.

Once again, we used a mixed effects GLM with the subject as a random effect (as each subject participated in two test conditions, each with 210 trials). The dependent variable was accuracy on labeling an end point of the continuum, which was coded as 1 or 0. The model included a main effect of trial number, a main effect of reading skill, and an interaction of the two. Trial number and reading skill were scaled and centered prior to modeling. The results of this analysis are provided in Table IX.

**TABLE IX.** Model of accuracy labeling continuum end points.

	*β*	SE	*p*
(Intercept)	1.672	0.067	<0.001
Trial	−0.103	0.022	<0.001
Reading skill	0.264	0.067	<0.001
Trial ∗ reading skill	−0.044	0.021	0.037

While there was a significant interaction of reading skill and trial number, the direction of this effect is actually opposite what we might have predicted—greater reading skill is associated with a more deleterious effect of trial number on accuracy. Inspection of our data reveals that this trend is strongly influenced by one particular subject in the dyslexic group who began with nearly chance accuracy and became more accurate over the course of the task. When we removed this subject from the model, the magnitude of the interaction effect more than halved (*β* went from −0.044 to −0.018) and the interaction was no longer significant (*p* = 0.397).

We also considered reaction time as a measure of the task engagement. Looking again at the continuum end points, we selected reaction times between 200 ms and 3 s (to remove spurious responses and outliers as in O'Brien *et al.*, 2018) and log-transformed these observations. The model of reaction time is given in Table X. Faster reaction time is associated with stronger reading skills (replicating O'Brien *et al.*, 2018). However, we did not detect a significant interaction between trial number and reading skill.

**TABLE X.** Model of reaction time labeling continuum end points.

	*β*	SE	*p*
Trial	0.007	0.004	0.053
Reading skill	−0.053	0.014	<0.001
Trial ∗ reading skill	0.003	0.004	0.49

As such, our results do not corroborate the idea that poor readers are especially prone to becoming disengaged, distracted, or tired during the task—at least by our proposed measures of engagement.

## IV. DISCUSSION

It is well established that phoneme categorization is related to reading skill, but there are a variety of explanations for this relationship. We considered how the frequency distribution from which stimuli are drawn during a standard phoneme labeling task might deferentially affect task performance in children with dyslexia versus task performance in typical readers. Indeed, some authors have posited that differences in psychophysical task performance may, in some situations, actually reflect a difference in an individual's sensitivity to task distributions (i.e., the distribution of the stimuli). Because our task did not appear to induce the sort of changes in psychometric functions that other authors have noted (such as slope), it is challenging to draw direct comparisons between our results and others (e.g., Clayards *et al.*, 2008; Vandermosten *et al.*, 2018). However, insofar as we detected some effect of the stimulus distribution statistics on aspects of task performance—where listeners draw a category boundary and a slight tendency to make more labeling errors of clear stimuli—we do not find evidence that the effect differs in children with dyslexia.

There are several reasons why we may not have seen the same effects of stimulus distribution as other authors: unlike in the study of distributional learning in children with and without reading disability by Vandermosten *et al.* (2018), we used a native-language contrast that may have been overlearned by our participants prior to our study. If this explanation were true, it would imply that children with dyslexia are entirely equipped to leverage statistical learning to establish phonetic categories from their natural environment (although we cannot rule out that they may do so to a lesser degree than their typically developing peers). However, there is evidence from the literature against this interpretation: even in typically developing children, identification functions produced by categorizing stop consonants do not fully resemble those of adults (Hazan and Barret, 2000, showed this in children aged 6–12 years, and McMurray *et al.*, 2018, largely replicated the finding in adolescents up to age 18 years). These findings argue that even native-language contrasts are unlikely to be fully learned by age 12 years, the oldest child in our study.

Another potential explanation for our differing results from previous reports is that our measurements may not have been sufficiently precise: Clayards *et al.* (2008) detected stimulus distribution effects on psychometric function shape for a native-language contrast in adults using eye-tracking to recover a time-series measure of looks to a closed set of choices displayed on a screen. We do not have access to a similarly fine-grained measure.

Our data set also allowed us to apply the modeling approach of Lieder *et al.* (2019) to investigate how previous stimulus presentations affect judgments about the current stimulus. We were able to detect effects of the previous two stimulus presentations but, critically, did not find that these effects interacted with reading skill. In other words, people with dyslexia show worse overall phoneme categorization performance but equivalent stimulus recency effects compared to people with typical reading skills. Our results are broadly consistent with the findings of Lieder *et al*, which showed that stimulus recency effects were similar in adults with and without dyslexia (albeit in a task involving the judgment of tone frequency differences).

For stimulus recency effects to be intact in children with dyslexia, it seems necessary that at least one aspect of sensory encoding is intact—if stimuli were not encoded with sufficiently high fidelity, it is difficult to imagine that children would be sensitive to differences between previous and current presentations. However, because we do not yet rigorously understand the neural or perceptual basis of categorical labeling, it is difficult to extend these results to understanding the quality of neural encoding involved in categorical decision-making. Thus, while our results indicate at *some* perceptual level that speech encoding is similar between groups, more work is needed to connect our findings to the broader debate about sensory encoding in dyslexia (Casini *et al.*, 2018; Goswami, 2011; Hancock *et al.*, 2017). At this point, our results can mainly be taken to contradict claims that adaptation or anchoring to recent stimuli is different in children with dyslexia (Ahissar *et al.*, 2006; Jaffe-Dax *et al.*, 2017; Krause, 2015; Nicolson and Fawcett, 2018; Perrachione *et al.*, 2016).

Finally, we tested whether children with dyslexia showed signs of increased fatigue during the task by analyzing their performance on relatively easy trials throughout the course of the experiment. Although individuals with dyslexia showed a tendency to make more errors on “easy” trials throughout the experiment, we did not find evidence to suggest that they were merely becoming less attentive over time as Messaoud-Galusi *et al.* (2011) did in a similar task. It is possible that our results differ because we allowed children a brief break (typically less than one minute) every 35 trials, whereas participants in the study by Messaoud-Galusi and colleagues adhered to a different schedule. Additionally, our participant demographics may have differed as many of our children are well-accustomed to computer games at home and at school. While we are, therefore, cautious to generalize our results broadly, we can conclude that task fatigue is unlikely to explain the patterns of categorical labeling we present here. This may be reassuring with regard to the large amount of literature on categorical labeling in individuals with dyslexia: while experimenters must remain vigilant of ways that overall decreased accuracy can bias measures of task performance (Roach *et al.*, 2004; Wichmann and Hill, 2001), our results suggest that a simple explanation of task engagement alone is unlikely to account for the entire relationship between reading and categorical labeling.

Considering our results and the current state of the field, we believe researchers are at an intriguing moment: there is compelling evidence that in certain experiments apparent deficits in groups of participants with dyslexia are well-explained by nonlinguistic and non-sensory mechanisms (Banai and Ahissar, 2004, 2006; Gabay *et al.*, 2015), and this framework has considerably more power to explain the diversity of deficits associated with reading disability than purely sensory or phonological models. Still, there are considerable gaps in this explanation: not only are there are experimental contexts where individuals with dyslexia appear to have no statistical learning deficit (Du and Kelly, 2013; Gabay and Holt, 2018; Gould and Glencross, 1990; Inácio *et al.*, 2018; Jiménez-Fernández *et al.*, 2011; Samara and Caravolas, 2017; Staels and Van den Broeck, 2015; perhaps reflecting ongoing vagueness in what “statistical learning” encompasses) but effect sizes in studies that do detect group differences are still too small to accurately separate most cases of dyslexia from typical reading (Lieder *et al.*, 2019; Vandermosten *et al.*, 2018).

Even if we take the view that reduced categorical labeling in struggling readers is entirely the consequence of impaired sensitivity to phonetic categories over the course of many years of language exposure, group separability would remain quite modest: the average effect size in categorical labeling studies is 0.66 (Noordenbos and Serniclaes, 2015), meaning that only 9.7% of individuals with dyslexia would fall below the 95% confidence interval of the control population. A further problem for the statistical learning hypothesis is that it often relies on an assumption that a very subtle impairment can cascade to have drastic effects on literacy by disrupting the development of typical phonological processing. However, a growing body of literature (Booth *et al.*, 2000; Calcus *et al.*, 2018; O'Brien *et al.*, 2018; Robertson *et al.*, 2009; Snowling *et al.*, 2019; Talcott *et al.*, 2000) suggests that a cascading model is inadequate: performance on psychophysical tasks can relate to reading skill separately from the proposed phonological processing mediation pathway. It may be that the categorical labeling task is an index of something far broader than phonological awareness, picking up on other aspects of developmental and linguistic experience.

Sharpening of category boundaries may be partially a result of reading experience itself. In this hypothesis, the acquisition of reading—and spelling skills, in particular—may create or reshape the representation of sound categories (Dich and Cohn, 2013). This hypothesis has been considerably understudied and would best be addressed via careful intervention studies in which category boundaries may be assessed before and after literacy training.

With that said, considering the results of the present study in conjunction with previous results from our group (O'Brien *et al.*, 2018; O'Brien *et al.*, 2019), we are hesitant to recommend the phoneme categorization task for future research. While these psychometric functions are reliably correlated with reading skill in our laboratory and many others, our ability to interpret them with regard to a mechanistic view of reading development is limited. As we have discussed, it is challenging to disambiguate sensory factors from broader aspects of language and cognitive development using these behavioral results. Further, it is not obvious how to extend behavioral results from this narrow experimental context to speech perception “in the wild” (Holt and Lotto, 2010). Seeing as this task has a long history as a probe for speech perception in reading-impaired populations dating back to the early 1980s (Noordenbos and Serniclaes, 2015), we are eager for researchers to develop new experimental paradigms that are guided by our field's maturing understanding of speech perception. For example, statistical methods and experimental tools have sufficiently advanced that researchers can consider time-series measures of behavioral responses (such as eye-tracking, as in McMurray *et al.*, 2018) and naturalistic, sentence-length stimuli incorporating talker variability. It is more possible than ever to create tasks that both resemble ethological speech perception and afford fine experimenter control, and we encourage researchers guided by mechanistic hypotheses to think beyond phoneme identification.

In summary, our results are consistent with the perspective that multiple causal routes relate performance on various behavioral and psychophysical measures to reading skill (Pennington *et al.*, 2012; Ziegler *et al.*, 2019). Under this model, deficits in learning category boundaries from speech sounds may be one of many factors that contribute to difficulties with reading—or, potentially, a consequence of developmental literacy challenges (Dich and Cohn, 2013). In light of this, we are most optimistic toward future research that explores how constellations of risk factors (Pennington *et al.*, 2012; Schatschneider *et al.*, 2016; Spencer *et al.*, 2014), including but not limited to reduced category learning, phonological processing, and sensitivity to the statistics of nonlinguistic stimuli, act in concert to determine a child's reading skill.

## Footnotes

^1^ See https://ReadingAndDyslexia.com (Last viewed 10/8/2020).

^2^ https://github.com/yeatmanlab/Context_Effects_Public (Last viewed 10/8/2020).

## References

[1] Ahissar, M. (2007). “ Dyslexia and the anchoring-deficit hypothesis,” Trends Cognit. Sci. 11(11), 458–465. 10.1016/j.tics.2007.08.015 17983834

[2] Ahissar, M. , Lubin, Y. , Putter-Katz, H. , and Banai, K. (2006). “ Dyslexia and the failure to form a perceptual anchor,” Nat. Neurosci. 9(12), 1558–1564. 10.1038/nn1800 17115044

[3] Amitay, S. , Ben-Yehudah, G. , Banai, K. , and Ahissar, M. (2002). “ Disabled readers suffer from visual and auditory impairments but not from a specific magnocellular deficit,” Brain 125(10), 2272–2285. 10.1093/brain/awf231 12244084

[4] Apfelbaum, K. S. , Hazeltine, E. , and McMurray, B. (2013). “ Statistical learning in reading: Variability in irrelevant letters helps children learn phonics skills,” Dev. Psychol. 49(7), 1348–1365. 10.1037/a0029839 22924367

[5] Banai, K. , and Ahissar, M. (2004). “ Poor frequency discrimination probes dyslexics with particularly impaired working memory,” Audiol. Neuro-Otol. 9(6), 328–340. 10.1159/000081282 15467286

[6] Banai, K. , and Ahissar, M. (2006). “ Auditory processing deficits in dyslexia: Task or stimulus related?,” Cereb. Cortex 16(12), 1718–1728. 10.1093/cercor/bhj107 16407480

[7] Blomert, L. , and Mitterer, H. (2004). “ The fragile nature of the speech-perception deficit in dyslexia: Natural vs. synthetic speech,” Brain Lang. 89(1), 21–26. 10.1016/S0093-934X(03)00305-5 15010233

[8] Boada, R. , Willcutt, E. G. , and Pennington, B. F. (2012). “ Understanding the comorbidity between dyslexia and attention-deficit/ hyperactivity disorder,” Top. Lang. Disord. 32, 264–284. 10.1097/TLD.0b013e31826203ac

[9] Boersma, P. , and Weenink, D. (2020). “ Praat: Doing phonetics by computer [Computer program].” Version 6.0.37, http://www.praat.org.

[10] Booth, J. R. , Perfetti, C. A. , MacWhinney, B. , and Hunt, S. B. (2000). “ The association of rapid temporal perception with orthographic and phonological processing in children and adults with reading impairment,” Sci. Stud. Read. 4(2), 101–132. 10.1207/S1532799XSSR0402_02

[11] Brainard, D. H. (1997). “ The psychophysics toolbox,” Spat. Vision 10(4), 433–436. 10.1163/156856897X00357 9176952

[12] Calcus, A. , Deltenre, P. , Colin, C. , and Kolinsky, R. (2018). “ Peripheral and central contribution to the difficulty of speech in noise perception in dyslexic children,” Dev. Sci. 21(3), e12558 10.1111/desc.12558 28256107

[13] Casini, L. , Pech-Georgel, C. , and Ziegler, J. C. (2018). “ It's about time: Revisiting temporal processing deficits in dyslexia,” Dev. Sci. 21(2), e12530 10.1111/desc.12530 28239921

[14] Clayards, M. , Tanenhaus, M. K. , Aslin, R. N. , and Jacobs, R. A. (2008). “ Perception of speech reflects optimal use of probabilistic speech cues,” Cognition 108(3), 804–809. 10.1016/j.cognition.2008.04.004 18582855 PMC2582186

[15] Dich, N. , and Cohn, A. (2013). “ A review of spelling acquisition: Spelling development as a source of evidence for the psychological reality of the phoneme,” Lingua 133, 213–229. 10.1016/j.lingua.2013.04.010

[16] Du, W. , and Kelly, S. W. (2013). “ Implicit sequence learning in dyslexia: A within-sequence comparison of first- and higher-order information,” Ann. Dyslexia 63(2), 154–170. 10.1007/s11881-012-0077-1 22996058

[17] Gabay, Y. , and Holt, L. L. (2018). “ Short-term adaptation to sound statistics is unimpaired in developmental dyslexia,” PLoS One 13(6), e0198146 10.1371/journal.pone.0198146 29879142 PMC5991687

[18] Gabay, Y. , Thiessen, E. D. , and Holt, L. L. (2015). “ Impaired statistical learning in developmental dyslexia,” J. Speech Lang. Hear. Res. 58(3), 934–945. 10.1044/2015_JSLHR-L-14-0324 25860795 PMC4490081

[19] Germanò, E. , Gagliano, A. , and Curatolo, P. (2010). “ Comorbidity of ADHD and dyslexia,” Dev. Neuropsychol. 35(5), 475–493. 10.1080/87565641.2010.494748 20721770

[20] Goswami, U. (2011). “ A temporal sampling framework for developmental dyslexia,” Trends Cognit. Sci. 15(1), 3–10. 10.1016/j.tics.2010.10.001 21093350

[21] Goswami, U. , Thomson, J. M. , Richardson, U. , Stainthorp, R. , Hughes, D. , Rosen, S. , and Scott, S. K. (2002). “ Amplitude envelope onsets and developmental dyslexia: A new hypothesis,” Proc. Natl. Acad. Sci. U.S.A. 99(16), 10911–10916. 10.1073/pnas.122368599 12142463 PMC125072

[22] Gould, J. H. , and Glencross, D. J. (1990). “ Do children with a specific reading disability have a general serial-ordering deficit?,” Neuropsychologia 28(3), 271–278. 10.1016/0028-3932(90)90020-O 2325839

[23] Hämäläinen, J. A. , Salminen, H. K. , and Leppänen, P. H. T. (2013). “ Basic auditory processing deficits in dyslexia,” J. Learn. Disabil. 46(5), 413–427. 10.1177/0022219411436213 22323280

[24] Hancock, R. , Pugh, K. R. , and Hoeft, F. (2017). “ Neural noise hypothesis of developmental dyslexia,” Trends Cognit. Sci. 21(6), 434–448. 10.1016/j.tics.2017.03.008 28400089 PMC5489551

[25] Hazan, V. , and Barret, S. (2000). “ The development of phonemic categorization in children aged 6–12,” J. Phonetics 28, 377–396. 10.1006/jpho.2000.0121

[26] Holt, L. , and Lotto, A. J. (2010). “ Speech perception as categorization,” Attent. Percept. Psychophys. 72(5), 1218–1227. 10.3758/APP.72.5.1218 PMC2921848 20601702

[27] Inácio, F. , Faísca, L. , Forkstam, C. , Araújo, S. , Bramão, I. , Reis, A. , and Petersson, K. M. (2018). “ Implicit sequence learning is preserved in dyslexic children,” Ann. Dyslexia 68(1), 1–14. 10.1007/s11881-018-0158-x 29616459

[28] Jaffe-Dax, S. , Frenkel, O. , and Ahissar, M. (2017). “ Dyslexics' faster decay of implicit memory for sounds and words is manifested in their shorter neural adaptation,” eLife 6, e20557 10.7554/eLife.20557 28115055 PMC5279949

[29] Jiménez-Fernández, G. , Vaquero, J. M. , Jiménez, L. , and Defior, S. (2011). “ Dyslexic children show deficits in implicit sequence learning, but not in explicit sequence learning or contextual cueing,” Ann. Dyslexia 61(1), 85–110. 10.1007/s11881-010-0048-3 21082295

[30] Kass, R. E. , and Raftery, A. E. (1995). “ Bayes factors,” J. Am. Stat. Assoc. 90, 773–795. 10.1080/01621459.1995.10476572

[31] Krause, M. B. (2015). “ Pay attention!: Sluggish multisensory attentional shifting as a core deficit in developmental dyslexia,” Dyslexia 21(4), 285–303. 10.1002/dys.1505 26338085

[32] Lieder, I. , Adam, V. , Frenkel, O. , Jaffe-Dax, S. , Sahani, M. , and Ahissar, M. (2019). “ Perceptual bias reveals slow-updating in autism and fast-forgetting in dyslexia,” Nat. Neurosci. 22(2), 256–264. 10.1038/s41593-018-0308-9 30643299

[33] Light, J. G. , Pennington, B. F. , Gilger, J. W. , and DeFries, J. C. (1995). “ Reading disability and hyperactivity disorder: Evidence for a common genetic etiology,” Dev. Neuropsychol. 11(3), 323–335. 10.1080/87565649509540623

[34] Maye, J. , Werker, J. F. , and Gerken, L. A. (2002). “ Infant sensitivity to distributional information can affect phonetic discrimination,” Cognition 82(3), B101–B111. 10.1016/S0010-0277(01)00157-3 11747867

[35] McMurray, B. , Aslin, R. N. , and Toscano, J. C. (2009). “ Statistical learning of phonetic categories: Insights from a computational approach,” Dev. Sci. 12, 369–378. 10.1111/j.1467-7687.2009.00822.x 19371359 PMC2742678

[36] McMurray, B. , Danelz, A. , Rigler, H. , and Seedorff, M. (2018). “ Speech categorization develops slowly through adolescence,” Dev. Psychol. 54(8), 1472–1491. 10.1037/dev0000542 29952600 PMC6062449

[37] Messaoud-Galusi, S. , Hazan, V. , and Rosen, S. (2011). “ Investigating speech perception in children with dyslexia: Is there evidence of a consistent deficit in individuals?,” J. Speech Lang. Hear. Res. 54(6), 1682–1701. 10.1044/1092-4388(2011/09-0261) 21930615 PMC3374927

[38] Nicolson, R. I. , and Fawcett, A. J. (2018). “ Procedural learning, dyslexia and delayed neural commitment,” in *Reading and Dyslexia*, edited by Lachmann T. and Weis T. ( Springer, Cham, Switzerland), pp. 235–269.

[39] Noordenbos, M. W. , and Serniclaes, W. (2015). “ The categorical perception deficit in dyslexia: A meta-analysis,” Sci. Stud. Read. 19(5), 340–359. 10.1080/10888438.2015.1052455

[40] O'Brien, G. E. , McCloy, D. R. , Kubota, E. C. , and Yeatman, J. D. (2018). “ Reading ability and phoneme categorization,” Nat. Sci. Rep. 8(1), 16842 10.1038/s41598-018-34823-8 PMC6237901 30442952

[41] O'Brien, G. E. , McCloy, D. R. , and Yeatman, J. D. (2019). “ Categorical phoneme labeling in children with dyslexia does not depend on stimulus duration,” J. Acoust. Soc. Am. 146(1), 245–255. 10.1121/1.5116568 31370631 PMC6639114

[42] Pennington, B. F. , Santerre-Lemmon, L. , Rosenberg, J. , MacDonald, B. , Boada, R. , Friend, A. , Leopold, D. R. , Samuelsson, S. , Byrne, B. , Willcutt, E. G. , and Olson, R. K. (2012). “ Individual prediction of dyslexia by single versus multiple deficit models,” J. Abnorm. Psychol. 121(1), 212–224. 10.1037/a0025823 22022952 PMC3270218

[43] Perrachione, T. K. , Del Tufo, S. N. , Winter, R. , Murtagh, J. , Cyr, A. , Chang, P. , Halverson, K. , Ghosh, S. S. , Christodoulou, J. A. , and Gabrieli, J. D. (2016). “ Dysfunction of rapid neural adaptation in dyslexia,” Neuron 92, 1383–1397. 10.1016/j.neuron.2016.11.020 28009278 PMC5226639

[44] Peters, L. , and Ansari, D. (2019). “ Are specific learning disorders truly specific, and are they disorders?,” Trends Neurosci. Educ. 17, 100115 10.1016/j.tine.2019.100115 31685130

[45] Ramus, F. , and Ahissar, M. (2012). “ Developmental dyslexia: The difficulties of interpreting poor performance, and the importance of normal performance,” Cognit. Neuropsychol. 29(1-2), 104–122. 10.1080/02643294.2012.677420 22559749

[46] Roach, N. W. , Edwards, V. T. , and Hogben, J. H. (2004). “ The tale is in the tail: An alternative hypothesis for psychophysical performance variability in dyslexia,” Perception 33(7), 817–830. 10.1068/p5207 15460509

[47] Robertson, E. K. , Joanisse, M. F. , Desroches, A. S. , and Ng, S. (2009). “ Categorical speech perception deficits distinguish language and reading impairments in children,” Dev. Sci. 12(5), 753–767. 10.1111/j.1467-7687.2009.00806.x 19702768

[48] Rosen, S. (2003). “ Auditory processing in dyslexia and specific language impairment: Is there a deficit? What is its nature? Does it explain anything?,” J. Phonetics 31(3-4), 509–527. 10.1016/S0095-4470(03)00046-9

[49] Saffran, J. R. , Aslin, R. N. , and Newport, E. L. (1996). “ Statistical learning by 8-month-old infants,” Science 274(5294), 1926–1928. 10.1126/science.274.5294.1926 8943209

[50] Samara, A. , and Caravolas, M. (2017). “ Artificial grammar learning in dyslexic and nondyslexic adults: Implications for orthographic learning,” Sci. Stud. Read. 21(1), 76–97. 10.1080/10888438.2016.1262865

[51] Schatschneider, C. , Wagner, R. K. , Hart, S. A. , and Tighe, E. L. (2016). “ Using simulations to investigate the longitudinal stability of alternative schemes for classifying and identifying children with reading disabilities,” Sci. Stud. Read. 201(1), 34–48. 10.1080/10888438.2015.1107072 PMC4732731 26834450

[52] Shaywitz, S. E. , Shaywitz, B. A. , Fletcher, J. M. , and Makuch, R. (1992). “ Evidence that dyslexia may represent the lower tail of a normal distribution of reading ability,” N. Engl. J. Med. 326,145–150. 10.1056/NEJM199201163260301 1727544

[53] Snowling, M. J. , Lervåg, A. , Nash, H. M. , and Hulme, C. (2019). “ Longitudinal relationships between speech perception, phonological skills and reading in children at high-risk of dyslexia,” Dev. Sci. 22, e12723 10.1111/desc.12723 30207641 PMC6492008

[54] Spencer, M. , Wagner, R. K. , Schatschneider, C. , Quinn, J. M. , Lopez, D. , and Petscher, Y. (2014). “ Incorporating RTI in a hybrid model of reading disability,” Learn. Disabil. Q. 37, 161–171. 10.1177/0731948714530967 25422531 PMC4240020

[55] Staels, E. , and Van den Broeck, W. (2015). “ No solid empirical evidence for the SOLID (serial order learning impairment) hypothesis of dyslexia,” J. Exp. Psychol. 41, 650–669. 10.1037/xlm0000054 25314161

[56] Stein, J. (2018). “ The current status of the magnocellular theory of developmental dyslexia,” Neuropsychologia 130, 66–77. 10.1016/j.neuropsychologia.2018.03.022 29588226

[57] Stevenson, J. , Langley, K. , Pay, H. , Payton, A. , Worthington, J. , Ollier, W. , and Thapar, A. (2005). “ Attention deficit hyperactivity disorder with reading disabilities: Preliminary genetic findings on the involvement of the ADRA2A gene,” J. Child Psychol. Psychiatry Allied Discip. 46(10), 1081–1088. 10.1111/j.1469-7610.2005.01533.x 16178932

[58] Stuart, G. W. , McAnally, K. I. , McKay, A. , Johnston, M. , and Castles, A. (2006). “ A test of the magnocellular deficit theory of dyslexia in an adult sample,” Cognit. Neuropsychol. 23(8), 1215–1229. 10.1080/02643290600814624 21049375

[59] Talcott, J. B. , Witton, C. , Mclean, M. F. , Hansen, P. C. , Rees, A. , Green, G. G. R. , and Stein, J. F. (2000). “ Dynamic sensory sensitivity and children's word decoding skills,” Proc. Natl. Acad. Sci. U.S.A. 97(6), 2952–2957. 10.1073/pnas.040546597 10688885 PMC16036

[60] Tallal, P. (1980). “ Auditory temporal perception, phonics, and reading disabilities in children,” Brain Lang. 9(2), 182–198. 10.1016/0093-934X(80)90139-X 7363063

[61] Tallal, P. , Miller, S. L. , Bedi, G. , Byma, G. , Wang, X. , Nagarajan, S. S. , Schreiner, C. , Jenkins, W. M. , and Merzenich, M. M. (1996). “ Language comprehension in language-learning impaired children improved with acoustically modified speech,” Science 271(5245), 81–84. 10.1126/science.271.5245.81 8539604

[62] Vandermosten, M. , Boets, B. , Luts, H. , Poelmans, H. , Golestani, N. , Wouters, J. , and Ghesquière, P. (2010). “ Adults with dyslexia are impaired in categorizing speech and nonspeech sounds on the basis of temporal cues,” Proc. Natl. Acad. Sci. U.S.A. 107(23), 10389–10394. 10.1073/pnas.0912858107 20498069 PMC2890794

[63] Vandermosten, M. , Boets, B. , Luts, H. , Poelmans, H. , Wouters, J. , and Ghesquière, P. (2011). “ Impairments in speech and nonspeech sound categorization in children with dyslexia are driven by temporal processing difficulties,” Res. Dev. Disabil. 32(2), 593–603. 10.1016/j.ridd.2010.12.015 21269803

[64] Vandermosten, M. , Wouters, J. , Ghesquière, P. , and Golestani, N. (2018). “ Statistical learning of speech sounds in dyslexic and typical reading children,” Sci. Stud. Read. 23(1), 116–127. 10.1080/10888438.2018.1473404

[65] Wagenmakers, E. J. (2007). “ A practical solution to the pervasive problems of *p* values,” Psychon. Bull. Rev. 14, 779–804. 10.3758/BF03194105 18087943

[66] Wichmann, F. A. , and Hill, N. J. (2001). “ The psychometric function: I. Fitting, sampling, and goodness of fit,” Percept. Psychophys. 63(8), 1293–1313. 10.3758/BF03194544 11800458

[67] Wolf, M. , and Bowers, P. G. (2000). “ Naming-speed processes and developmental reading disabilities: An introduction to the special issue on the double-deficit hypothesis,” J. Learn. Disabil. 33(4), 322–324. 10.1177/002221940003300404 15493094

[68] Ziegler, J. C. (2008). “ Better to lose the anchor than the whole ship,” Trends Cognit. Sci. 12(7), 244–245. 10.1016/j.tics.2008.04.001 18515174

[69] Ziegler, J. C. , and Goswami, U. (2005). “ Reading acquisition, developmental dyslexia, and skilled reading across languages: A psycholinguistic grain size theory,” Psychol. Bull. 131(1), 3–29. 10.1037/0033-2909.131.1.3 15631549

[70] Ziegler, J. C. , Perry, C. , and Zorzi, M. (2019). “ Modeling the variability of developmental dyslexia,” in *Developmental Dyslexia Across Languages and Writing Systems* ( Cambridge University Press, Cambridge, UK), Chap. 16, p. 350.

